# GLOBAL CLIMATE ATTITUDES IN AGING ADULTS: THE ROLES OF SCIENTIFIC TRUST AND LITERACY

**DOI:** 10.1093/geroni/igae098.2321

**Published:** 2024-12-31

**Authors:** Bryce Van Vleet, Heather Fuller

**Affiliations:** North Dakota State University, Fargo, North Dakota, United States; North Dakota State University, Fargo, North Dakota, United States

## Abstract

Prior research has established that people with less scientific literacy are more distrustful of science. However, this link is not well understood in older age or in relation to climate change perceptions. Aging adults play an important role in and are particularly vulnerable to the effects of climate change. This study investigates relationships between scientific literacy and trust with perceptions of climate change utilizing the 2020 Wellcome Global Monitor. 33,075 adults aged 50-99 (M=62.47) from 113 countries were interviewed about their views on science. We investigated whether scientific literacy and trust in science were predictors of climate change: a) understanding, b) major threat perception, and c) denial. Multiple regression analyses indicated trust in science and scientific literacy significantly predicted climate change understanding and threat perception, but not denial. Given conceptual distinctions between scientific illiteracy and scientifically literate mistrust, we investigated whether trust in science mediated the relationship between scientific literacy and climate change attitudes in midlife and older adulthood. Bootstrapped mediation models indicated trust in science partially mediated associations between scientific literacy and both understanding and threat perception. Findings suggest bolstering confidence in both scientific literacy and trust in science may help aging populations better understand and anticipate impacts of climate change. Results can help interventionists prepare aging populations for an increasingly warming world. Findings also extend the existing understanding of trust in science and scientific literacy as important processes in how older individuals perceive their environmental and politicized contexts.

